# Effect of the estrus cycle stage on the establishment of murine endometriosis lesions

**Published:** 2018-05

**Authors:** Kiandokht Kiani, Mansoureh Movahedin, Hossein Malekafzali, Faramarz Mirfasihi, Seyedeh Nargess Sadati, Ashraf Moini, SeyedNasser Ostad, Reza Aflatoonian

**Affiliations:** 1 *Valiasr Reproductive Health Research Center, Tehran University of Medical Sciences, Tehran, Iran.*; 2 *Department of Endocrinology and Female Infertility, Reproductive Biomedicine Research Center, Royan Institute for Reproductive Biomedicine, ACECR, Tehran, Iran. *; 3 *Department of Anatomical Sciences, Faculty of Medical Sciences, Tarbiat Modares University, Tehran, Iran.*; 4 *Department of Epidemiology and Biostatistics, School of Public Health, Tehran University of Medical Sciences, Tehran, Iran.*; 5 *Department of Pathology, Massoud Clinical Laboratory, Tehran, Iran.*; 6 *Department of Traditional Pharmacy, School of Traditional Medicine, Tehran University of Medical Sciences, Tehran, Iran.*; 7 *Department of Obstetrics and Gynecology, School of Medicine, Tehran University of Medical Sciences, Tehran, Iran.*; 8 *Department of Toxicology and Pharmacology, School of Pharmacy, Tehran University of Medical Sciences, Tehran, Iran.*

**Keywords:** Endometriosis, Animal models, Transplantation, Autologous, Estrus cycle

## Abstract

**Background:**

Establishment of a standardized animal endometriosis model is necessary for evaluation of new drug effects and for explaining different ethological aspects of this disease. For this purpose, we need a model which has more similarity to human endometriosis.

**Objective:**

Our objective was to establish an autologous endometriosis mouse model based on endogenous estrogen level and analyze the influence of estrus cycle on the maintenance of endometriotic lesions.

**Materials and Methods:**

In this experimental study, endometriotic lesions were induced in 52 female NMRI mice by suturing uterine tissue samples to the abdominal wall. The transplantation was either performed at proestrus/estrus or at metestrus/diestrus cycles. Urine-soaked beddings from males and also male vasectomized mice were transferred to the cages to synchronize and maintenance of estrus cycle in female mice. The mice were sacrificed after different transplantation periods (2, 4, 6 or 8 wk). The lesions size, macroscopic growth, model success rate, histological and immune-histochemical analyses were assessed at the end.

**Results:**

From a total of 200 tissue samples sutured into the peritoneal cavity, 83 endometriotic lesions were confirmed by histopathology (41.5%). Model success rate for proestrus/estrus mice was 60.7% vs. 79.2% for metestrus/diestrus mice. The endometriotic lesions had similar growth in both groups. Number of caspase-3, Ki67-positive cells and CD31-positive micro vessels were also similar in endometriotic lesions of two groups.

**Conclusion:**

If we maintain the endogenous estrogen levels in mice, we can induce endometriosis mouse model in both proestrus/estrus and metestrus/diestrus cycle without any significant difference.

## Introduction

Retrograde menstruation to the peritoneal cavity has been considered as the cause of endometriosis by Sampson’s theory (1). So far, researchers have established different animal endometriosis models in both primates and non-primates based on the mentioned theory (2-3). Primates like baboon exhibit spontaneous menstruation similar to human beings. However, they are expensive and their handling is rather a difficult task. Therefore, rodent endometriosis models are used in preclinical research due to their low costs, convenient handling and genetic similarity (2). Establishment of a reliable and standardized animal model of endometriosis is necessary for the evaluation of new drug effects and for explaining different aspects of pathology or etiology of this disease. For this purpose, we need a model which has more similarity to human endometriosis. 

Induction of endometriosis in rodents can be achieved by transferring the uterine tissue samples surgically or by injection into the peritoneal cavity (4-7). In the majority of previous studies, either the endometrium layer or all layers of uterine tissue is implanted at proestrus phase and in some other studies, the estrus cycle is not mentioned (6). 

Endometriosis is an estrogen-dependent disease and endometriotic lesions will have enough growth in presence of a high level of estrogen (10). In previous studies, most researchers used the endometrium or uterine tissues at proestrus or estrus phase (6, 11, 12). However, proestrus is a very short phase, and endometriotic lesions do not have enough time to be exposed to enough estrogen level. The estrogen level is rapidly decreased in late proestrus and estrus as compared with metestrus and diestrus phases (13). There is only one study that found the endometriotic lesions in proestrus and estrus is fast to heal compared to metestrus/diestrus rats. They showed the slow regression of endometriotic lesions when the endometrial tissue was implanted during the metestrus and diestrus cycle. Their findings suggest that it is better to use animals in the stage of metesrus/diestrus for endometriosis lesions (14). 

One important issue in the establishment of the endometriosis model for preclinical studies is to follow up the endometriotic lesions during the drug administration. Some researchers used high-resolution ultrasound in order to follow up the lesions (15, 11), some researchers opened the abdominal wall of rats by surgery every 2 or 4 wk and they excluded the rats with regressed lesions (16-18) and some studies used laparoscopy for mice (14, 19). But unfortunately, we did not have access to high-resolution ultrasound or laparoscopy for mice in our country. In addition, opening the abdominal wall induces the inflammatory reactions and changes the peritoneal cavity environment and also it is dangerous for the mouse. Therefore, we need a reliable method for preparing endometriosis mouse model.

In this study, we tried to establish an autologous endometriosis mouse model based on endogenous estrogen level and analyze the influence of estrus cycle on the maintenance of endometriotic lesions in different time points.

## Materials and methods

**Animals**

Fifty-two female NMRI (Naval Medical Research Institute) mice aged 10-16 wk with a body weight of 24-30 gr were used for experiments. The animals were housed in groups of five per cage under standard laboratory conditions within a temperature-controlled environment on a 12 hr light/dark cycle. They had free access to water and standard pellet food. The manuscript was prepared according to the ARRIVE Guidelines (20). 

**Figure 1:**
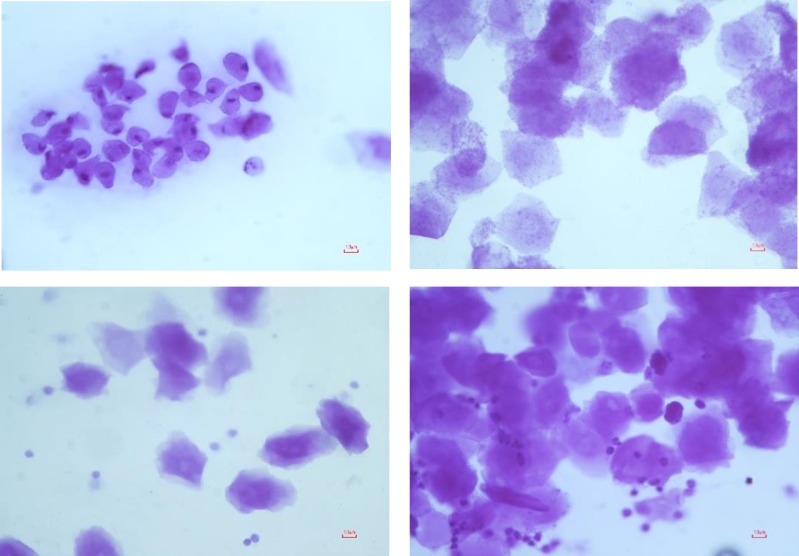
Cytological assessment of vaginal smears stained by Crystal Violet (A) Proestrus: epithelial cells-mostly rounded but some cells showing early stages of cornification of approaching estrus (B) Estrus: large cornified cells in clumps (C) Metestrus: leucocytes with smaller numbers of non-nucleated epithelial cells (D) Diestrus: mainly leucocytes with small number of nucleated epithelial and cornified cells. Scale bars: 10 µm

The housing of female mice without any exposure to male pheromones leads to the cessation of cycling. Urine-soaked beddings from males and also male vasectomized mice were transferred to the females' cages to synchronize and maintenance of estrus cycle in female mice, a phenomenon referred to as Whitten effect (21). The vaginal lavage method was used for the identification of the estrus cycle stage of individual animals. For this purpose, 50-100 µl of sterile ddH2O was carefully pipetted into the vagina and then transferred on a glass slide for examination under a light microscope (22) (Figure 1). The transplantation was either performed at proestrus/estrus or at metestrus/diestrus cycles.

**Endometriosis model**

The mice were randomly divided into two groups based on their cycle stage on the day of surgical induction of endometriotic lesions: group 1 (proestrus or estrus, n=28), group 2 (metestrus or diestrus, n=24). All mice were anesthetized using an intraperitoneal injection of 100 mg/kg Ketamine (Alfasan, Woerden-Holland) and 10 mg/kg Xylazine 2% (Alfasan, Woerden-Holland), diluted at 1:10 (v/v) in sterile distilled water (23). Subsequently, a 2-cm vertical midline incision was made 0.5-1.0 cm rostral to the vaginal opening, and both the uterine horns were exposed. The left uterine horn was ligated between the cervix and ovary and was removed and transferred into a sterile glass Petri dish containing 2-3 mL of sterile phosphate buffer solution (PBS). By inserting one blade of small scissors under a loop microscope (Olympus-SZ60, Tokyo, Japan), the horn was opened longitudinally and divided into 2 mm segments with a sterile biopsy punch in the glass Petri dish (5). These small uterine segments were sutured (without removing myometrium) onto the upper and lower parts of the inner surface of the abdominal wall (two to the right and two to the left side). Finally, the peritoneum and skin were closed with absorbable sutures. The mice were allowed to recover on a warm plate. All the operations were performed by a single researcher. The mice were analyzed after different implantation periods. Every 2 wk, some mice underwent an autopsy. 

**Macroscopic evaluation**

For macroscopic evaluation, we assessed mean of lesions size and mean of macroscopic growth degree. Lesion size was calculated by multiplying the width and length of the lesions (expressed in mm^2^) measured by caliper and then mean of lesions size was measured per mouse. Macroscopic growth degree was calculated in accordance to Quereda and colleagues (24) with some modifications and classified in the following way: “degree 0-implantation disappears or absence of the cystic form; degree I-implantation forms a vesicle with a diameter smaller than 2 millimeters or solid form; degree II-implantation is cystic with fluid and its diameter is 2-4.5 mm; and degree III-vesicle with a diameter higher than 4.5 mm”. Cumulative macroscopic growth degree was calculated in each mouse. Degrees 0 or 1 were considered as an inadequate growth of lesions and degrees 2 or 3 were attributed to the adequate growth of lesions.

**Histopathological examination and model success rate**

The formalin-fixed endometriotic lesions were embedded in paraffin.sections (5-µm thickness) were stained with hematoxylin and eosin and examined under a light microscope. The endometriosis was diagnosed by identification of endometrial glandular tissue and stroma. The total model success rate was measured by dividing the total number of retrieved endometriotic lesions (confirmed by the pathology (by the total number of implanted uterine tissues multiplied by 100, which is expressed as a percentage. The individual peritoneal implant rate was defined as the number of retrieved endometriotic lesions (confirmed by pathology) divided by the number of implanted uterine tissues per animal multiplied by 100, which is expressed as a percentage.

**Evaluation of persisting epithelium in endometrial autografts**

Pathological evaluation of uterine autografts was performed by a semi-quantitative method, namely histopathological score (25) as following: “a well-preserved epithelial layer was scored 3, a moderately preserved epithelium with leukocyte infiltrate was scored 2, a poorly preserved epithelium (occasional epithelial cells only) was scored 1, and cases with no epithelium were scored 0”. Then mean histopathological score was calculated in each mouse.

**Immunofluorescent analysis for apoptosis, cell proliferation, and vascularization of endometriotic lesions**

Indirect immunofluorescence was used for immunohistochemistry. The endometriotic lesions were removed and fixed in formalin. They were then sectioned into 5 µm thick slices. The endometriotic lesion sections were fixed for 10 min in ice-cold acetone and were thereafter rehydrated in PBS containing 0.1% Triton X-100 for 5 min. The tissue was permeabilized and blocked in blocking solution containing 1% BSA/10% normal goat serum/0.3M glycine in 0.1% PBS-Tween for 1 hr. The sections were incubated overnight at 4^o^C with the following primary antibodies: rabbit polyclonal Caspase 3 (Abcam, ab4051) diluted 1:100 (for detection of apoptotic cells); rabbit polyclonal to Ki67 (Abcam, ab15580) diluted 1:100 (for detection of proliferating cells); rabbit polyclonal to Cd31 (Abcam, ab28364) diluted 1:100 (for detection of micro vessels in endometriotic lesions). 

All dilutions were done in PBS containing 0.2% Triton X-100, 1% BSA and 2% normal goat serum. The sections were subsequently washed with PBS and incubated with goat-anti-rabbit Phycoerythrin (PE) conjugated (1:400; Invitrogen) as secondary antibody for 1 hr. at room temperature. In addition, double staining was performed using DAPI (KPL) 1:5000. The same procedure was used for negative controls without using of primary antibodies. 

Immunohistochemistry images were analyzed using the Image J software (http://rsbweb.nih.gov/ij/). The immune reactivity of the antibodies was visualized and photographed with an invert fluorescent microscope (Nikon, Japan) (200X). The fraction (percentage) of Ki67, CD31, and Caspase-3-positive cells was measured two sections per one lesion from each mouse at different estrus stages. All data were normalized to the area of endometriotic lesion section or the number of cells present in the lesion.

**Ethical consideration**

All the experiments were approved by the Ethics Committee of University of Medical Sciences, Tehran, Iran. The experiments were in agreement with Iran's Ministry of Health guidelines for the care and use of laboratory animals.

**Statistical analysis**

Data were expressed as mean ± standard deviation (S.D.). The data was first analyzed for normal distribution by SPSS software (Statistical Package for the Social Sciences, version 21.0, SPSS Inc, Chicago, Illinois, USA). Statistical analyses were performed using a Student’s *t*-test or Mann Whitney u-test for nonparametric data. p<0.05 were considered to be significant.

## Results

**Macroscopic results**

36 mice (69.2%) had visible lesions at the day of sacrifice. Two wk post-induction, there were small visible macroscopic lesions on the right or left peritoneum wall. Six wk post-implantation, all mice (100%) in metestrus/diestrus and 90.9% of mice at proestrus/estrus developed at least one cystic endometriotic lesion. The lesions appearance often was cystic with a homogenous surface. Our results showed that the factor `estrus cycle` did not significantly influence the size and macroscopic growth degree of endometriotic lesions at all observation time points (Table I). All endometriotic lesions were at degrees 2 or 3, which were considered as the adequate growth of lesions.

**Histopathological examination and implant success rate**

**Figure 2:**
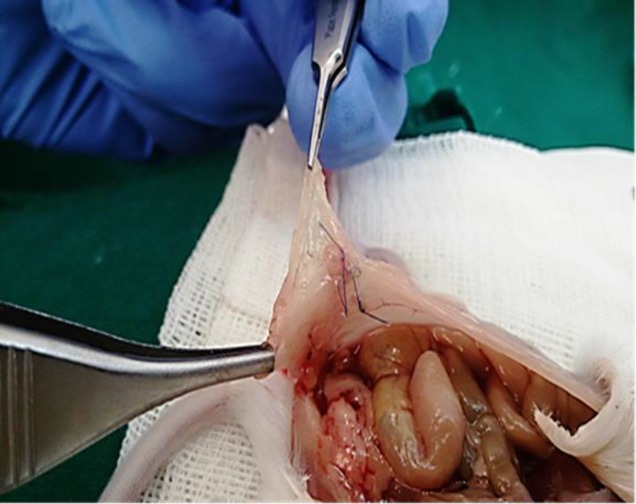
The cystic endometriotic lesion on the right lower quadrant of peritoneum, 6 wk after implantation

**Figure 3:**
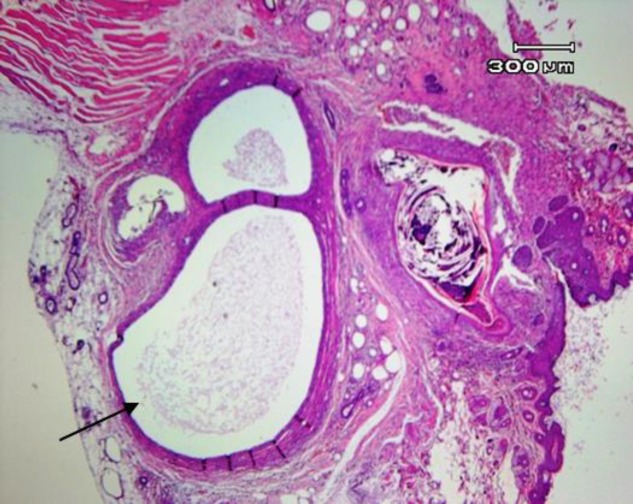
H&E stained section of a cystic endometriotic peritoneal lesion, 6 wk after implantation, Magnification: 40×.

Typical histologic criteria of endometriotic lesions, including the presence of glandular epithelium and stromal elements, were confirmed in all lesions. Figure 2 shows the morphology and appearance of a peritoneal lesion after 6 wk of implantation. The endometriotic lesions of mice included a zone composed of 2 to 6 layer cells of endometrial stroma bearing scattered endometrial glands and an inner layer consisting of a single row of mostly columnar epithelial cells with occasional areas of pseudo-stratification. The lumen was filled with an amorphous eosinophilic material (Figure 3). 

Histological characteristics of the remaining right uterine horn and the right ovary were normal without any evidence of endometriosis. Only acute inflammatory changes were observable in the perimetrium layer of uterine horn in some mice. From a total of 200 tissue samples sutured into the peritoneal cavity (109 tissue samples at proestrus/estrus and 91 tissues at metestrus/diestrus), 83 endometriotic lesions were confirmed by histopathology (overall model success rate of 41.5%). The model success rate for proestrus/estrus mice (phase1) was 60.7% vs. 79.2% for metestrus/diestrus (phase2) ones (p=0.229). The factor `estrus cycle` did not significantly influence the histopathological scores of endometriotic lesions (Table I).

**Apoptosis, cell proliferation and vascularization in endometriotic lesions**

**Figure 4:**
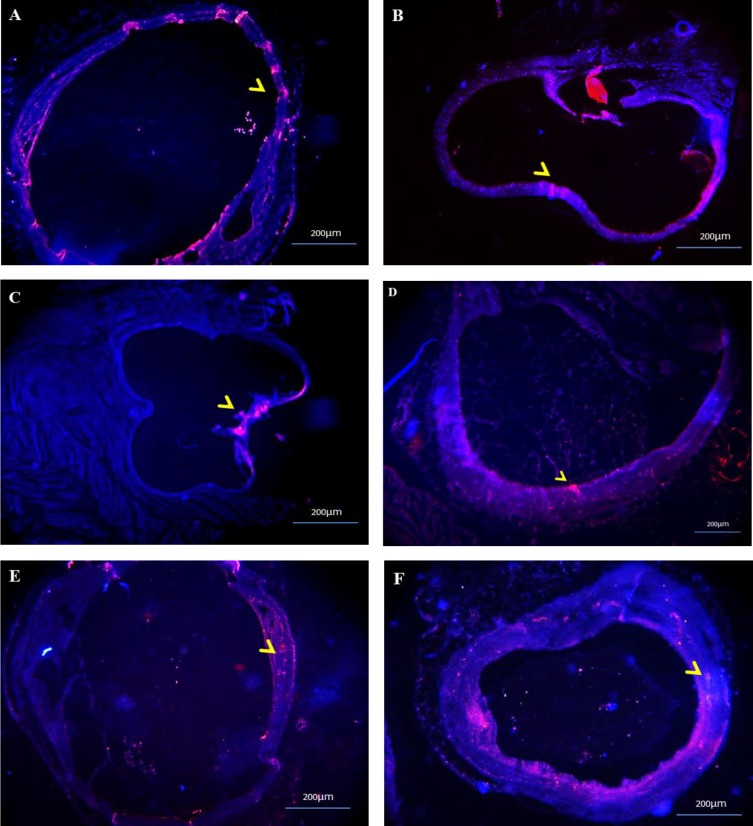
(A–F) Immunofluorescent sections of endometriotic lesions at proestrus/estrus (A, C, E) or at metestrus/diestrus stages (B, D, F), by fixation of uterine tissue samples to the peritoneal wall; Sections were stained with DAPI to identify cell nuclei (blue), an antibody against the apoptosis marker Caspase-3 (red) (A-B); and an antibody against the proliferation marker Ki67 (red) (C-D); an antibody against CD31 for the detection of the microvascular endothelium (red) (E-F). Magnification: ×200.

**Figure 5:**
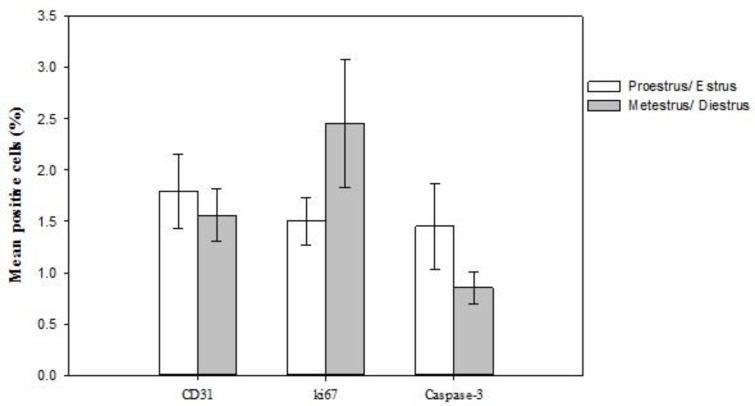
Caspase-3, Ki67 positive cells (%) and CD 31 positive micro vessels (%) in peritoneal endometriotic lesions at proestrus/ estrus (white bars) or metestrus/estrus (Grey bars) stages at operation day. Mean ± SEM.

Figure 4 shows a sample of immunocytochemistry sections from endometriotic lesions, indicating the expression of Caspase 3, ki67, and CD31 proteins. We could not found any significant difference in caspase-3-positive apoptotic cells of endometriotic lesions between two estrus cycles. In addition, the number of Ki67-positive stromal and glandular cells in endometriotic lesions and angiogenesis or vascularization of endometriotic lesions, which was indicated by CD31-positive micro vessels, was similar between two groups (Figure 5).

**Table I:** Results of autologous endometriosis models induced into the peritoneal cavity of 52 NMRI mice according to different estrus stages at operation day

**Observation time points**	**Implants success rate (%)**		**Mean** **macroscopic growth degree**		**Mean lesion size (mm** ^2^ **)**		**Mean** **lesion score**	
	Proestrus / Estrus (n= 7)	Metestrus / Diestrus (n= 6)	Proestrus / Estrus (n= 7)	Metestrus / Diestrus (n= 6)	Proestrus / Estrus (n= 7)	Metestrus / Diestrus (n= 6)	Proestrus / Estrus (n= 7)	Metestrus / Diestrus (n= 6)
2 wk (n=13)	25 ± 28.8	36.1 ± 32.8	0.7 ± 1.5	1.1 ± 1.3	7.5 ± 2.1	4.2 ± 1.2	2.2 ± 0.5	2.3 ± 0.7
p-value	0.529		0.741		0.121		1.00	
4 wk (n=13)	50 ± 43.3	22.8 ± 29.1	2.1 ± 0.3	2 ± 1	5 ± 1.2	5.7 ± 0.6	2.2 ± 0.8	1.8 ± 0.3
p-value	0.219		0.864		0.427		0.453	
6 wk (n=13)	35 ± 36.5	58 ± 13.3	2.4 ± 0.9	2.3 ± 0.8	7.6 ± 6.7	6.9 ± 2.3	2.05 ± 1	2.4 ± 0.8
p-value	0.173		0.840		0.851		0.562	
8 wk (n=13)	39.3 ± 49.7	68.3 ± 19.1	1.8 ± 0.3	1.9 ± 0.54	6.7 ± 1.2	7.7 ± 4.8	2.4 ± 0.09	2.5 ± 0.4
p-value	0.379		0.396		0.439		0.598	

Data are expressed as Mean ± S.D.

Mann Whitney u-test

## Discussion

At present study, we used an autologous endometriosis mouse model without ovariectomy or any estradiol supplementation in order to mimic the human endometriosis and also we assessed the individual implant success rate of endometriosis as well as the change of endometriotic lesions during different stages of the estrous cycle in mice. Our results showed similar model success rate, similar macroscopic and histopathologic characteristics of endometriotic lesions at both estrus stages.

Endometriosis is an estrogen-dependent disease and endometriotic lesions will have enough growth in presence of a high level of estrogen (10). In previous studies, most researchers used the endometrium or uterine tissues at proestrus or estrus phase (6, 11-12). At late proestrus and estrus phase, the endometrial thickness of mice is higher than the other stages (26). This thickness may facilitate the endometrial sampling and provide enough tissue for suturing to the peritoneal wall. However, proestrus is a very short phase, and endometriotic lesions do not have enough time to be exposed to enough estrogen level. The proestrus phase of estrus cycle starts by increasing the 17-β-estradiol levels. This increment leads to indirect stimulation of gonadotropin-releasing hormone neurons in the hypothalamus and releasing luteinizing hormone and follicle-stimulating hormone into the circulation (22). In human, this phase is considered as follicular phase of the menstrual cycle (27). The peak in follicle-stimulating hormone levels stimulates the ovulation and estrus cycle will start. During estrus cycle, 17-β-estradiol decreases and prolactin has peak (22). In the other hand, metestrus/diestrus phases in mice are most comparable with the menstrual phase in humans and may be a suitable model for human endometriosis. Metestrus phase and increase amount of progesterone occur simultaneously and it is compatible with the start of human luteal phase (28). As progesterone levels begin to increase and there is a small spike in 17-β- estradiol levels in response to the activation of corpus luteum (28). Finally, entry into diestrus in mice happens and circulating progesterone levels peak. This phase is related to the human late luteal phase (29). Following a sharp decline in the level of progesterone, regression of the corpus luteum occurs (30). 

Our results showed that the factor `estrus cycle` did not significantly influence the endometriosis size and macroscopic growth degree of endometriotic lesions in all four time transplantation periods. This finding is contrary to findings of Uchida and colleagues who found a correlation between the volume of endometriosis and the estrous cycle (14). Previous researchers have shown that endometriosis is a dynamic disorder causing spontaneous changes in lesion appearance, progression and regression over time (31). Endometriotic cysts regress with low estrogen levels, especially at estrus cycle, and reappear when the level of estrogen rises, especially at high estrogen levels (32). The estrogen level is rapidly decreased in late proestrus as compared with metestrus and diestrus phases (14). It means that maintenance of periodical sexual stages at mouse is more important than specific stage like proestrus or metestrus.

There was no significant difference in the mean histopathological scores between proestrus/estrus or metestrus/diestrus mice. Endometriotic lesions in all mice groups showed good or moderately preserved epithelial integrity with preserved well-ordered stroma. The presence of glandular epithelium is a hallmark and a diagnostic criterion for human endometriosis (25). This finding is similar to Wood and colleagues who showed that luminal epithelium proliferation, which was assessed by PCNA immunohistochemistry, was similar in different estrus stages (26). It means that this model can provide suitable and acceptable endometriotic lesions and can be comparable with the moderate stage of human endometriosis. In addition, all estrus stages showed adequate macroscopic growth of lesions according to Quereda and colleagues (24). 

In the present study, we also compared apoptosis, proliferation, and angiogenesis using immune histochemistry among two estrus cycle at operation day. We detected no difference with respect to cleaved Caspase-3-positive apoptotic cells, the number of neither Ki67-positive stromal and glandular cells nor angiogenesis or vascularization of endometriotic lesions, which was indicated by CD31-positive micro vessels in four time periods. It means that endometriosis model at both estrus stages prepare similar proliferation and similar angiogenesis for endometriotic lesions. Similar Ki67positive cells was also found in women with and without the endometriosis in three studies (33). In addition, similar apoptotic cells at endometriotic lesions in both groups suggest the reason for similar incidence and similar survival of endometriotic tissues at both estrus stages. Anti-apoptosis signaling and the acceleration of proliferation are typical molecular properties associated with the survival of endometriotic tissues (34-35). It is well established that endometriotic lesions need to gain a new blood supply to survive in ectopic sites, making angiogenesis essential for the development and establishment of endometriosis (36). Menstrual cycle phase has a significant effect on vessel segment length, both within each region and within uterus layers. However, we did not assess these findings in relation to estrous stage classification. Future studies should assess these factors in discrete estrus phases.

In most previous studies, either the endometriosis was implanted at proestrus phase or authors did not mention the estrus cycle (6). Furthermore, different methods such as homologous or heterologous were used. The lack of consistency with respect to estrogen supplementation, low and variable (30-50%) or unreported peritoneal implant success rates as well as model success rate are some other limitations of these studies (2, 37, 38). Establishment of a reliable and standardized animal model of endometriosis is necessary for the evaluation of new drug effects and for explaining different aspects of pathology or etiology of this disease. For this purpose, we need a model which has more similarity to human endometriosis. Our findings showed that we can use endometrium tissues at any estrus stages for autologous endometriosis mouse model.

## Conclusion

In conclusion, these results show that the estrous cycle at operation day has no effect on the establishment and macroscopic or histological changes of ectopic endometriotic lesions at autologus endometriosis mouse model. If we maintain the endogenous estrogen levels in mice, we can induce endometriosis mouse model in both proestrus/estrus and metestrus/diestrus cycle without any significant difference.

## Note

***This article extracted from Ph.D. thesis. (Kiandokht Kiani)***
